# Cardioprotective effects of PKG activation by soluble GC activator, BAY 60-2770, in ischemia-reperfusion-injured rat hearts

**DOI:** 10.1371/journal.pone.0180207

**Published:** 2017-07-03

**Authors:** Kyung Hye Lee, So-Ra Lee, Haneul Cho, Jong Shin Woo, Jung Hee Kang, Yun-Mi Jeong, Xian Wu Cheng, Woo-Shik Kim, Weon Kim

**Affiliations:** Department of Cardiovascular of Internal Medicine, Kyung Hee University Hospital, Kyung Hee University, Seoul, Republic of Korea; Emory University, UNITED STATES

## Abstract

Soluble guanylate cyclase (sGC) has been suggested as a therapeutic target for cardiac ischemia-reperfusion (IR) injury. Until now, the molecular mechanism of BAY 60–2770, a sGC activator, in cardiac IR injury has not been assessed. To identify the cardioprotective effects of BAY 60–2770 in IR-injured rat hearts, IR injury was established by occlusion of LAD for 40 min and reperfusion for 7 days, and the effects of BAY 60–2770 on myocardial protection were assessed by echocardiography and TTC staining. 5 nM and 5 μM of BAY 60–2770 were perfused into isolated rat hearts in a Langendorff system. After 10- or 30-min reperfusion with BAY 60–2770, cGMP and cAMP concentrations and PKG activation status were examined. Hearts were also perfused with 1 μM KT5823 or 100 μM 5-HD in conjunction with 5 nM Bay 60–2770 to evaluate the protective role of PKG. Mitochondrial oxidative stress was investigated under hypoxia-reoxygenation in H9c2 cells. In IR-injured rat hearts, BAY 60–2770 oral administration reduced infarct size by TTC staining and improved left ventricular function by echocardiography. Tissue samples from BAY 60-2770-perfused hearts had approximately two-fold higher cGMP levels. BAY 60–2770 increased PKG activity in the myocardium, and the reduced infarct area by BAY 60–2770 was abrogated by KT-5823 in isolated myocardium. In H9c2 cardiac myoblasts, hypoxia-reoxygenation-mediated mitochondrial ROS generation was diminished with BAY 60–2770 treatment, but was recovered by pretreatment with KT-5823. BAY 60–2770 demonstrated a protective effect against cardiac IR injury via mitoKATP opening and decreased mitoROS by PKG activation. BAY 60–2770 has a protective effect against cardiac IR injury via mitoKATP opening and decreased mitoROS by PKG activation. These results demonstrated that BAY 60–2770 may be used as a therapeutic agent for cardiac IR injury.

## Introduction

Myocardial ischemic-reperfusion (IR) injury is induced through quick restoration of blood flow, resulting in robust reactive oxygen species (ROS) generation and damage or dysfunction of the cardiac tissue [1]. IR-injured cardiac myocytes have some limitations under pathological conditions, including reduced nitric oxide (NO) generation and oxidized heme in soluble guanylate cyclase (sGC). sGC is an important enzyme in the cardiovascular system and is expressed in vascular endothelial cells [2], smooth muscle cells [3] and cardiac myocytes [4, 5]. sGC stimulators such as YC-1 and BAY 41–2272 trigger the generation of cyclic 3’,5-guanosine monophosphate (cGMP) without NO binding [6, 7]. sGC activators target NO-insensitive oxidized, heme-free sGC, which protects sGC from proteasomal degradation [8].

NO-independent sGC activators have emerged as valuable tools for elucidating the physiopathology of the NO-cGMP pathway. BAY 58–2770 (cinaciguat) and HMR-1766 (ataciguat) have been studied in the progression of cardiovascular disease due to their roles as activators of oxidized sGC [8, 9]. Moreover, the therapeutic potency of the sGC activator BAY 60–2770 (4-[[[4-carboxybutyl] [2-[5-fluoro-2-[[4’-[trifluoromethyl] biphenyl-4-yl] methoxy] phenyl] ethyl] amino] methyl] benzoic acid) has been elucidated in nitrate-tolerant arteries [10, 11], obesity [12, 13], platelet activity [14] and asthma [15]. Despite studies investigating the protective role of BAY 60–2770 in coronary artery occlusion [16], few studies have focused on mitochondrial ROS in cardiac IR injury.

Mitochondria impairment has been discussed as a key target of ischemic heart disease. Oxygen deprivation during ischemia decreases intracellular ATP and pH leading to an increase in intracellular and mitochondrial Ca^2+^ levels, which causes ROS production from mitochondrial electron transfer complexes I and III [17]. After the onset of reperfusion, the ROS burst worsens mitochondrial dysfunction and damage to the mitochondrial membrane [18]. Outer membrane permeabilization leads to cytochrome c release and subsequent apoptosis, and massive oxidative stress-induced mitochondrial inner membrane permeability contributes to the opening of the mitochondria permeability transition pore (mPTP) [18]. Thus, mitochondria dysfunction is a central mediator of damage involving cell death in cardiac IR injury [19].

To demonstrate the cardioprotective effects of the potent sGC activator, BAY 60–2770, which potentiates NO/GC/cGMP signaling, we treated IR-injured rat hearts with BAY 60–2770. Here, we investigated the effects of BAY 60–2770 on the cGMP-PKG pathway and on mitochondrial ROS regulation.

## Materials and methods

### Materials

The following pharmacological agents were used in this study. BAY 60–2770 (a gift from Bayer Pharma AG, Germany); K^+^ channel blocker, 5-HD (5-hydroxydecanoate-sodium salt, Enzo, NY, USA); potent and selective inhibitor of PKG, KT5823 (2,3,9,10,11,12-hexahydro-10R-methoxy-2,9-dimethyl-1-oxo-9S, Cayman Chemical, MI, USA); mitochondria uncoupler, CCCP (carbonyl cyanide 3-chlorophenylhydrazone, Sigma, St. Louis, MO, USA).

### Experimental animals

Healthy male normotensive 8-9-week-old Sprague-Dawley rats weighing 250–300 g were obtained from Orient. Bio Inc. (Seongnam, Korea) and were used after a 1-week acclimation period under standard laboratory conditions. All experimental procedures were approved by the Kyung Hee University Hospital Animal Experimentation Committee. The care and use of all animals was in accordance with our institutional guidelines and conformed to the Guide for the Care and Use of Laboratory Animals published by the US National Institutes of Health (NIH Publication No. 85–23, revised 1996).

### In vivo IR model and measurement of infarction size

Animals were randomly assigned to one of three groups: the normal control group (Nor; n = 10), the IR group (IR; n = 5) or the BAY 60-2770-treated IR group (BAY; n = 6). BAY 60–2770 (5 mg/kg) or the same volume of vehicle was administered orally 30 min before the onset of the operation. The concentration of BAY 60–2770 (5 mg/ml) used in each experiment was determined according to preliminary experiments (data not shown). Following oral administration, rats were anesthetized by intraperitoneal injection (0.9 ml/100 g body weight) of ketamine (50 mg/ml) and xylazine (20 mg/ml) at 6.25:1. Myocardial ischemia was produced by occlusion of the left anterior descending coronary artery (LAD) using a slipknot (5–0 silk). After 40 min of ischemia, the ligature was released, and blood flow was restored. Left ventricular (LV) function was analyzed with echocardiography after seven days of reperfusion. The heart was removed, and the LV was sliced transversely into 2-mm slices, photographed, and stained with 1% 2,3,5-triphenyltetrazolium chloride (TTC) at 37°C for 15 min. Tissue slices were immersed in 10% formalin to enhance contrast between stained and unstained tissues. Infarct zone areas were calculated for each animal from computerized color images using NIH Image software (National Institutes of Health, USA).

### Echocardiography

Transthoracic echocardiography (Sonos 5500, Philips Ultrasound, Bothell, WA, USA) was performed to evaluate LV function as described previously [20]. Under anesthesia, the chest was shaved, and two-dimensional echocardiography was performed using our echocardiographic system with a 15-MHz probe. M-mode echocardiography of the LV at the papillary muscle level was performed, guided by two-dimensional short axis images. Parameters were averaged from three cardiac cycles. The LV ejection fraction (%EF) and fractional shortening (%FS) were automatically calculated by the echocardiographic system.

### Ex vivo Langendorff perfusion experiment

For analyzing cGMP and cAMP concentration, nine rats were randomly divided into three groups: IR injury with DMSO vehicle (IR, n = 3), IR injury with 5 nM Bay (5 nM, n = 3) and with 5 μM Bay (5 μM, n = 3). After aortic cannulation, hearts were mounted on a Langendorff system (AD Instruments Ltd., Chalgrove, UK) and allowed to stabilize for 30 min in modified Krebs-Henseleit solution containing 118 mM NaCl, 4.7 mM KCl, 1.9 mM CaCl_2_, 1.2 mM MgSO_4_, 25 mM NaHCO_3_, 1.2 mM KH_2_PO_4_, 10.1 mM glucose, 0.5 mM Na-EDTA and oxygenated with 95% O_2_/5% CO_2_, pH 7.4 at 37°C and constant pressure (80 mmHg). Global ischemia was maintained for 30 min followed by 10 or 30 min reperfusion.

To determine the cGMP and cAMP concentrations and PKG activity, hearts were perfused with BAY60-2770 (5 nM or 5 μM) for 10 min. To demonstrate the role of PKG in BAY 60-2770-induced myocardial protection, hearts were perfused with KT-5823 (1 μM) or 5-HD (100 μM) in addition to BAY 60–2770 for 30 min after an ischemic period (n = 6). At the end of the reperfusion period, three hearts from each group were perfused with 5 ml 0.1% Evans blue dissolved in Krebs-Henseleit solution, followed by a brief perfusion with Krebs-Henseleit solution alone. Sliced tissues were stained with triphenyltetrazolium chloride (TTC) at 37°C for 20 min. Infarct and risk areas were measured using Image J (NIH, version 1.47t). All other tissues were homogenized for analyzing PKG activity and for Western blot analysis.

### cGMP and cAMP measurements

After perfusion with BAY using the Langendorff apparatus, snap frozen heart tissues including RV and LV were weighed and homogenized. cGMP and cAMP measurements were performed according to the manufacturer’s instructions using a cGMP complete ELISA kit and a cAMP complete ELISA kit (Enzo Life Science, Farmingdale, NY, USA). The amount of cGMP and cAMP are presented as pmol/mg of heart.

### PKG activity assay and Western blot analysis

Cardiac PKG activity was measured with the CycLex Cyclic GMP-dependent protein kinase (cGK/PKG) Assay Kit (Cyclex, MBL International Corp, Nagoya, Japan) from tissue homogenates. Activity was measured according to the manufacturer’s instructions. Spectrophotometic absorbance was measured at 450 nm. Results were normalized as per mg of protein, and relative activity is presented.

Homogenates of whole hearts were used for Western blotting. Proteins were separated by 10% SDS-PAGE and transferred to a PVDF membrane. Non-specific binding was blocked with 5% skim milk in 1X Tris-buffer containing 0.1% Tween-20 for an hour. Blots were incubated overnight with primary antibodies (1:000, Cell Signaling Technology, MA, USA) and visualized with anti-rabbit or anti-mouse horseradish peroxidase-conjugated secondary antibody (1:5000, Vector Laboratories, Burlingame, CA, USA) for 1 hour. Blots were developed with ECL chemi-luminescence detection reagent (Bio-Rad, CA, USA).

### Cardiac myocyte culture and mitochondrial ROS determination

Rat embryonic cardiac myoblast H9c2 cells (ATCC, VA, USA) were used between passage numbers 10 and 25. Cells were cultured in Dulbecco’s Modified Eagle’s Medium (DMEM, Gibco, CA, USA), supplemented with 10% fetal bovine serum, 100 U/ml of penicillin, and 100 U/ml of streptomycin at 37°C in a humidified atmosphere of 5% CO_2_. Hypoxic conditions were achieved by flushing with 95% N_2_/5% CO_2_ using a hypoxia chamber (Billups-Rothenberg, Inc., Del Mar, CA, USA). The sealed chamber was placed into a 37°C incubator for 14 hr following treatment with BAY 60–2770 or KT-5823 in media containing 0.5% FBS. After incubation, cells received fresh media and were restored in 95% air/5% CO_2_ for a 6-hr reoxygenation period.

Mitochondrial superoxide production was measured by fluorescence microscopy using 2 μM of MitoSOX Red (Life Technologies, NY, USA) for 30 min at 37°C. H9c2 cells were treated with MitoTracker Green (Life Technologies) to stain mitochondria. After a brief counter staining with DAPI, cells were analyzed by confocal microscopy LSM700 (Carl Zeiss, Germany) at 400 X magnification. CCCP (2 μM) was used as a positive control for inducing mitochondrial ROS. Cells were also examined using a multi-mode microplate fluorescence reader Spark 10M (Tecan, Männedorf, Switzerland) at wavelength Ex/Em 510/580 nm.

### Statistical analysis

All data were analyzed using GraphPad Prism v5.01 (GraphPad Software, Inc., CA, USA). Values are presented as the mean ± SD. Statistical analyses were performed with one-way ANOVA (*post-hoc*; Newman-Keuls tests). Values of p <0.05 were considered statistically significant.

## Results

### BAY 60–2770 attenuated cardiac IR injury

**Fig 1:**
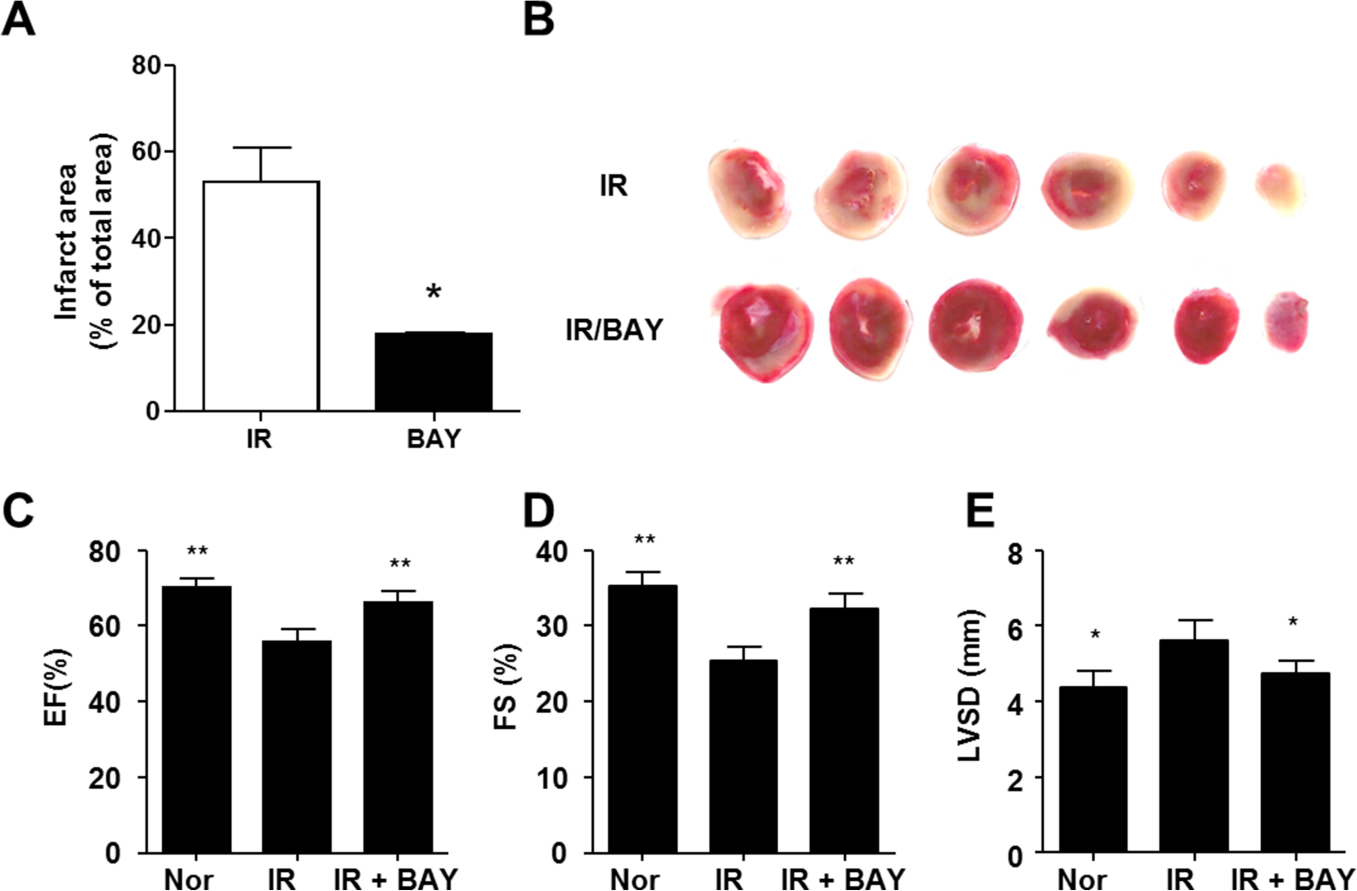
BAY 60–2770 reduces myocardial infarction in a rat IR injury model. Seven days after reperfusion, the infarct area was visualized using TTC staining from IR-injured and BAY 60–2770 pre-treated hearts (A). Representative TTC images from Nor (n = 10), IR (n = 5), BAY (n = 6) (B). *p < 0.05 between IR and BAY groups. Echocardiographic analysis is shown as percent of EF (C), FS (D), and LVDD (mm, E). *p <0.05, **p <0.005 vs. IR.

The effect of BAY 60–2770 on myocardial protection in IR injury was assessed by TTC staining and echocardiography. In IR-injured rat hearts, treatment with BAY60-2770 limited infarct size (53.08 ± 11.31% in IR vs. 17.91 ± 0.66% in BAY) by TTC staining (Fig 1A and 1B). Echocardiography showed that the reduced EF (70.1 ± 2.3% vs. 55.8 ± 3.2% in the Nor and IR groups, respectively) by IR injury was recovered in the BAY 60–2770 treatment group (66.2 ± 2.9% in BAY) (Fig 1C). Recovery of FS (%) and LVSD (mm) was apparent in the BAY treated group compared with the IR group (Fig 1D and 1E). Detailed results of transthoracic echocardiography are shown in Table 1. No significant difference in HR was found between the IR and BAY groups.

**Table 1:** Echocardiographic analysis.

	Nor (n = 10)	IR (n = 5)	BAY (n = 6)
**HR**	327.3 ± 55.7	294.0 ± 23.1	315.7 ± 68.1
**IVS**	0.95 ± 0.12	0.93 ± 0.14	1.02 ± 0.10
**PW**	1.02 ± 0.11	1.05 ± 0.15	1.08 ± 0.09
**LVMI**	0.91 ± 0.09	0.97 ± 0.09	0.97 ± 0.09
**LV diastolic D**	6.79 ± 0.58	0.74 ± 0.76	7.24 ± 0.71
**LV systolic D**	4.39 ± 0.42*	5.62 ± 0.54	4.74 ± 0.34*
**LVEDV**	0.72 ± 0.17	0.97 ± 0.27	0.87 ± 0.24
**LVESV**	0.21 ± 0.06*	0.43 ± 0.11	0.23 ± 0.05*
**EF**	70.1 ± 2.3**	55.8 ± 3.2	66.2 ± 2.9**
**FS**	35.3 ± 1.7**	25.4 ± 1.9	32.2 ± 2.1**

Values represent mean ± SEM.

*p<0.05

^**^p < 0.01 (compared with IR)

HR, heart rate; IVS, interventricular septal thickness in diastole; PW, posterior wall thickness in diastole; LVMI, left ventricular mass index; LV diastolic D, left ventricle diastolic dimension; LV systolic D, left ventricle systolic dimension; LVEDV, LV end diastolic volume; LVESV, LV end systolic volume; EF, ejection fraction; FS, fractional shortening.

### BAY 60–2770 activates PKG in cardiac IR injury

To investigate the novel cardiac protective mechanism of BAY 60–2770, 5 nM or 5 μM of Bay 60–2770 were perfused into isolated rat hearts in the Langendorff system. After 10 min reperfusion with BAY 60–2770, cGMP and cAMP concentrations and PKG activation statuses were examined. Tissue samples from BAY 60-2770-perfused hearts had cGMP levels about 2-fold higher than IR (17.2 ± 7.6 in IR vs. 32.8 ± 4.6 in 5 nM vs. 38.4 ± 10.0 pmol/mg tissue in 5 μM) without any alteration in cAMP concentration.

**Fig 2:**
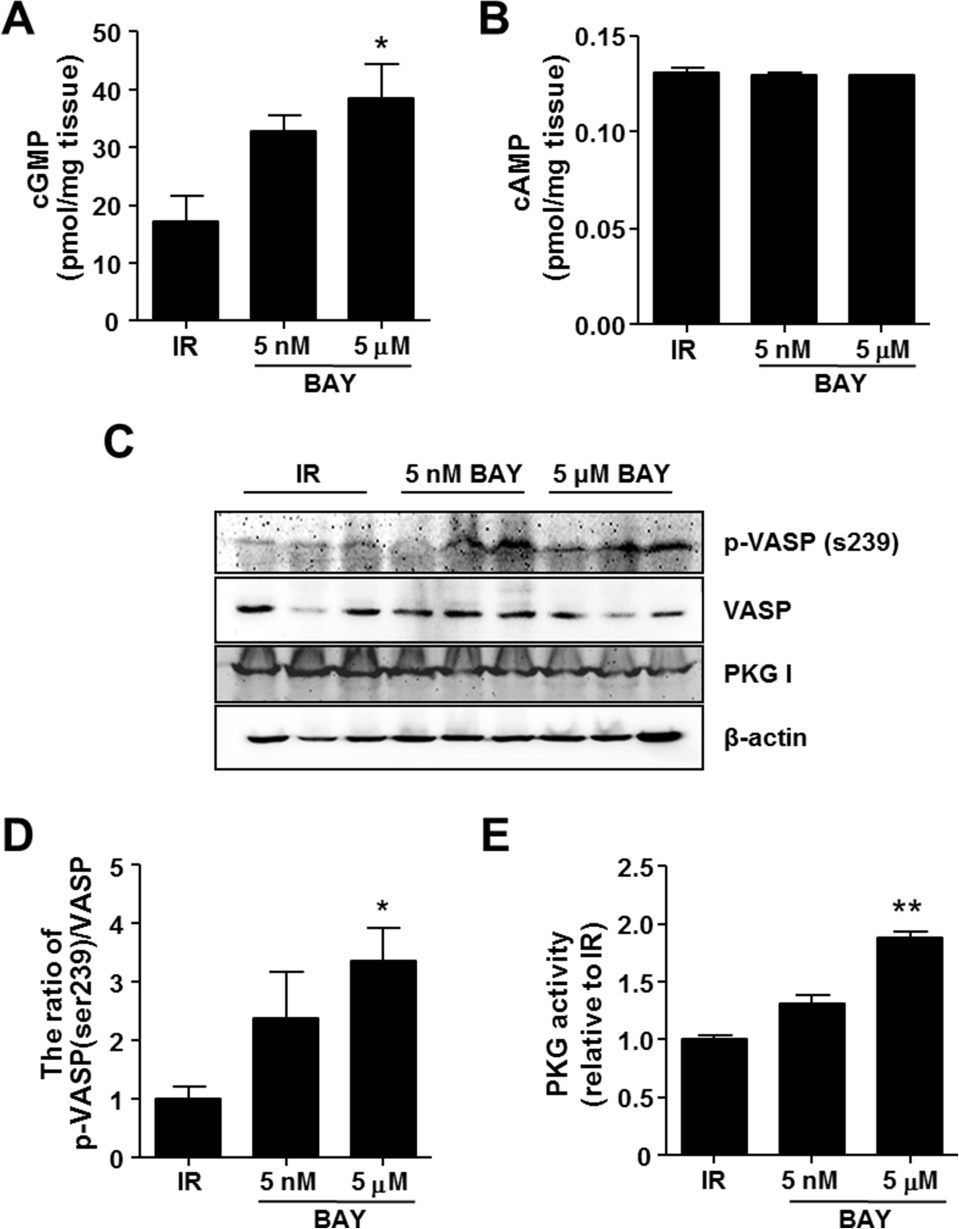
BAY 60–2770 activates PKG in isolated cardiac tissue. BAY 60–2770 was perfused at 5 nM or 5 μM for 10 min in a Langendorff system. cGMP (A) and cAMP (B) concentrations in tissue homogenates were measured. Western blot analysis was used to determine VASP phosphorylation at ser239 (C), and the ratio of phospho-VASP to VASP was analyzed (D). PKG activity was examined using the CycLex cGK/PKG Assay (E). All the experiments were performed duplicated or triplicated in 3 animals from each group. *p <0.05, **p <0.005 vs. IR.

To examine whether increased cGMP activates PKG in IR injury, phosphorylation of serine 239 in VASP, a substrate of PKG, was evaluated. BAY 60–2770 elevated VASP phosphorylation without PKG expression alteration (Fig 2C and 2D). Interestingly, the expression level of total VASP decreased in 5 μM BAY treated heart, while the levels were not affected in 5 nM BAY. The activity of PKG in the myocardium after reperfusion was also increased by BAY 60–2770 treatment (Fig 2E). Taken together, these results suggest that BAY 60–2770, an activator of sGC, activates PKG through cGMP production in IR-injured myocardium.

### PKG-mediated BAY 60-2770-induced protection

**Fig 3:**
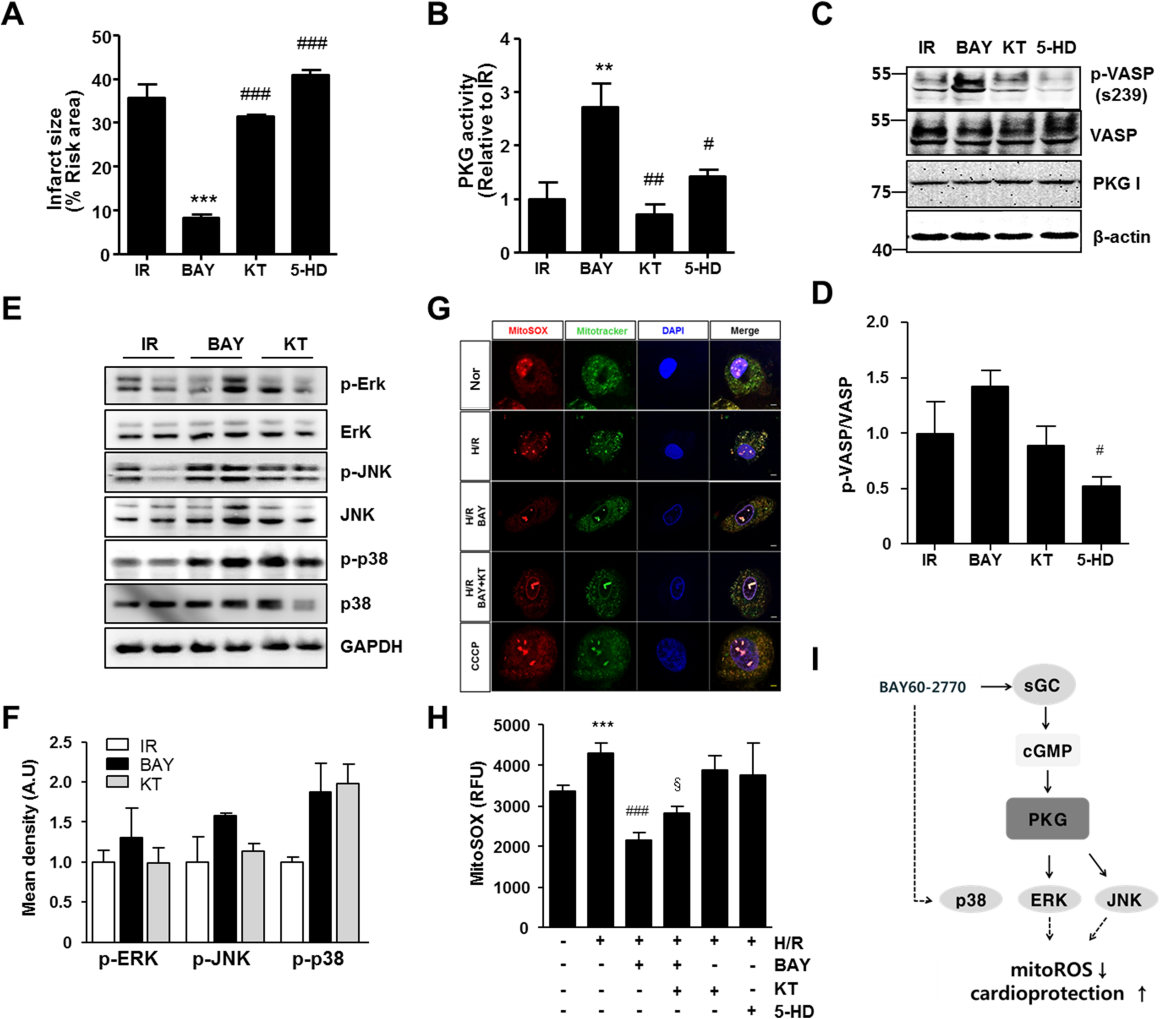
PKG mediates BAY 60-2770-induced cardioprotection. BAY 60–2770 (5 nM) was perfused with KT-5823 (1 μM) or 5-HD (100 μM) for 30 min after global ischemia. The percent of infarct area to risk area was determined from hearts from 3 animals in each group (A). PKG activity (B), phosphorylation of VASP (C, D) and MAPKs (E, F) were analyzed duplicated in 3 animals from each group. **p <0.005, ***p <0.001 vs. IR. # p <0.05, ## p <0.005, ### p <0.001 vs. BAY. Hypoxia/reoxygenation (H/R) injury was established by 14 hr of hypoxia and 6 hr reoxygenation. BAY 60–2770 and KT-5823 were treated during H/R injury in H9c2 cells, and cells were stained with MitoSOX red and counter stained with Mitotracker green and DAPI. Confocal microscopic images were taken under 400 X magnification (Scale bar: 5 μm) (G). Relative fluorescence unit (RFU) of MitoSOX was measured with microplate fluorescence reader (Ex/Em 510/580 nm). Mean values of at least quardruple samples are shown (H). A schematic diagram of postulated molecular pathway of BAY 60–2770 in cardiac IR injury (I). PKG plays a role in lowering mitochondrial ROS formation and limiting infarct size in BAY 60–2770 treated IR injured heart. The requirement of MAPKs activation on the PKG-mediated cardioprotection remains to be elucidated (dashed arrow). **p <0.005 vs. Nor. ### p <0.001 vs H/R. §p <0.05 vs. BAY.

We next tested whether PKG-mediated BAY 60-2770-induced protection in IR injury. KT5823 (1 μM) was perfused with 5 nM BAY 60–2770 for 30 min, and the infarct area was examined. PKG activity and immunoblots were analyzed with homogenates from BAY 60-2770-perfused global ischemic hearts. As we noticed from our in vivo experiments, BAY 60–2770 ameliorated the IR injury in the ex vivo Langendorff model. The infarct area was decreased by BAY 60–2770. KT-5823 co-treated hearts had a significant deterioration in infarct size (Fig 3A). The anti-infarct effect of BAY 60–2770 was completely blocked by treatment with PKG inhibitor and 5-HD, a selective mitoKATP channel inhibitor, suggesting that PKG and mitoKATP channel opening is required for protection by BAY 60–2770. In addition, PKG activity (Fig 3B) and VASP phosphorylation on ser239 (Fig 3C and 3D) were increased by BAY 60–2770; both were completely inhibited by KT-5823 co-administration without alteration of PKG expression (Fig 3C). 5-HD pretreatment limited PKG activation (Fig 3B, 3C and 3D). Western blot analysis (Fig 3E) demonstrated that PKG is a mediator of ERK and JNK phosphorylation, but it did not alter p38 phosphorylation. These results suggest that mitoKATP channel opening is required for activation of PKG, which mediates ERK and JNK phosphorylation in BAY 60–2770 treated cardiac IR injury.

### BAY 60–2770 reduces mitochondrial superoxide

MitoKATP channel opening is known to inhibit mitochondrial superoxide generation [21]. To investigate whether BAY 60–2770 regulates mitochondrial ROS under reoxygenation stress, hypoxia-reoxygenated (H/R) H9c2 cells were pre-treated with BAY 60–2770 and stained with MitoSOX Red (Fig 3G). H/R exposure-induced mitochondrial superoxide was abrogated with BAY 60–2770. Pretreatment with KT-5823 reduced this response, suggesting that PKG mediated BAY 60–2770 induces mitochondrial ROS inhibition (Fig 3G and 3H). The relative fluorescence unit examined from multi-mode reader determined that KT-5823 or 5-HD without BAY 60–2770 treatment had no significant differences on MitoSOX in H/R injury (Fig 3H).

## Discussion

Cardiac IR injury increased mitochondrial oxygen radical production and further enhanced oxidative cellular damage. Increased generation of ROS has been suggested to be a major contributor to the pathogenic mechanisms of cell death and cardiac IR injury [19]. Mitochondrial ROS generation and mitochondrial matrix Ca^2+^ overload converge to induce opening of the mitochondrial permeability transition pore [17]. Therefore, reducing mitochondrial ROS production upon reperfusion is an appealing therapeutic target for minimizing IR injury. Because IR injury has pathological conditions that reduce NO generation and oxidize heme in sGC, several sGC activators have been developed and used as agents to protect against IR injury.

We evaluated the role of recently developed BAY 60–2770 in regulating mitochondrial ROS production and limiting infarct size in cardiac IR injury. As previously reported, under oxidative stress conditions, enhanced levels of ROS inhibit ATP synthesis, and this inhibition prevails over inhibition of the two proteins, ROS activate mitoKATP channel and UCP, which in turn dissipate ΔΨ, thereby preventing further intensive ROS formation [22, 23]. MitoKATP channel has been implicated in cardiac protection during H_2_O_2_-induced cardiomyocyte apoptosis [24], ischemic preconditioning (IPC) [25], post IR arrhythmia [26], and IR injury [27, 28]. MitoKATP channel opening prevents excessive ROS accumulation under IR injury by promoting mild uncoupling and thus preventing mitochondrial ROS generation, rather than by enhancing the cellular ROS removal system [29]. Ozcan and coworkers showed that opening of the mitoKATP channel protects the isolated cardiac mitochondria by attenuating oxidative stress after reoxygenation [30]. Bice *et al*. reported the infarct limiting and cGMP induction cardioprotective effects of sGC activator BAY 60–2770 [16]. We were further interested in the role of BAY 60–2270 in mitochondrial ROS and assumed that sGC-cGMP–PKG regulates mitochondrial ROS and cardiac IR injury.

To the best of our knowledge, the present study is the first to demonstrate the infarct limiting mechanism of BAY 60–2770. BAY60-2770-induced PKG activation was confirmed using an ELISA based PKG activity assay and Western blot analysis with phosphor-specific VASP at ser239, a substrate of PKG in the *ex vivo* global ischemia-reperfusion model, demonstrating that BAY60-2770 had a direct effect on cardiac tissue and myocytes. We observed that KT-5823, a selective PKG inhibitor, aggravated the infarct limiting and mitochondrial ROS inhibition effects of BAY 60–2770. 5-HD, a mitoKATP channel opening inhibitor and inhibitor of ischemic preconditioning, has detrimental effects on BAY 60–2770 treated IR injury. PKG activation by BAY 60–2770 was abolished with 5-HD, implicating PKG interplay with mitoKATP (Fig 3B and 3C). We speculate that mitoKATP opening helps to maintain PKG in the active mode and exerts additive infarct limiting effects.

Survival protein kinases, especially members of the MAPK family, are activated during both the ischemic preconditioning and myocardial reperfusion periods [31]. These survival kinases were believed to activate ROS generation under opening of mitochondrial K(ATP) channel [32, 33], which activates EKR [33] and p38 MAPK [32] through ROS generation in the protection pathway. The paradox of JNK is that it has both protective and detrimental aspects of cardioprotection [31]. In our study, three kinds of MAPKs were activated with BAY 60–2770, and ERK and JNK phosphorylation were decreased with KT-5823 treatment, suggesting that PKG activates both of ERK and JNK in BAY 60–2770 administered hearts. In previous experiments using sildenafil in IR injury, PKG-dependent ERK phosphorylation was considered as indispensable pathway of cardioprotection [34]. Because of the lack of evidence of the role of MAPKs, further studies are needed to elucidate the exact interplay of PKG and MAPKs with BAY 60–2770 in cardiac IR injury. The schematic diagram of postulated molecular pathways of BAY 60–2770 in cardiac IR injury was shown in Fig 3I.

Study limitations should be considered. First, here we have studied the risk-at-area at the short-term (30 minutes) after reperfusion injury. Secondly, although the effects of KT5823 or 5-HD on BAY-mediated cardioprotective actions were investigated by ex vivo and in vitro experiments, an in vivo experiment was not designed to explore both drugs’ effects. Third, our major concern is that we did not determine the area at risk (AAR) for the in vivo coronary arteries ligation experiment. These measurements are critical for assessing the consistency of coronary occlusion and serve as a critical control. Because infarction after I/R injury is variable in many rodent experiments and the histological approach is strongly affected by precise sequence and conditions, lack of AAR determination in our in vivo IR injury is a major limitation to be considered. Further studies will be needed to investigate these issues.

Furthermore, mitochondrial ROS generation under hypoxia-reoxygenation stress in H9c2 cardiac myocytes was reduced with BAY 60–2770 treatment and was reversed with KT-5823, suggesting that PKG mediates inhibition of mitochondrial ROS production. These findings suggest that increasing sGC and related PKG activity ameliorates mitochondrial dysfunction including ROS generation triggered by IR injury. We emphasize the role of BAY 60–2770 in regulating sGC-cGMP-PKG-ERK and interplay with mitoKATP against cardiac IR injury.

In conclusion, BAY 60–2770 demonstrated a protective effect against cardiac IR injury via PKG activation and decreased ROS by mitoKATP opening. BAY 60–2770 may be used as a therapeutic agent for cardiac IR injury.
